# Manipulation of Shallow-Trap States in Halide Double Perovskite Enables Real-Time Radiation Dosimetry

**DOI:** 10.1021/acscentsci.3c00691

**Published:** 2023-09-11

**Authors:** Yumin Wang, Gaoyuan Chen, Zibin Zhu, Haoming Qin, Liangwei Yang, Duo Zhang, Yingguo Yang, Menglin Qiu, Ke Liu, Zhifang Chai, Wanjian Yin, Yaxing Wang, Shuao Wang

**Affiliations:** † State Key Laboratory of Radiation Medicine and Protection, School for Radiological and Interdisciplinary Sciences (RAD-X) and Collaborative Innovation Center of Radiation Medicine of Jiangsu Higher Education Institutions, Soochow University, Suzhou 215123, China; ‡ College of Energy, Soochow Institute for Energy and Materials Innovations (SIEMIS), Jiangsu Provincial Key Laboratory for Advanced Carbon Materials and Wearable Energy Technologies, Soochow University, Suzhou 215006, China; § Jiangsu Key Laboratory of Micro and Nano Heat Fluid Flow Technology and Energy Application, School of Physical Science and Technology, Suzhou University of Science and Technology, Suzhou, 215009, China; ∥ Shanghai Synchrotron Radiation Facility (SSRF), Zhangjiang Lab, Shanghai Advanced Research Institute, Shanghai Institute of Applied Physics, Chinese Academy of Sciences, Shanghai 201204, China; ⊥ Key Laboratory of Beam Technology of Ministry of Education, College of Nuclear Science and Technology, Beijing Normal University, Beijing 100875, China

## Abstract

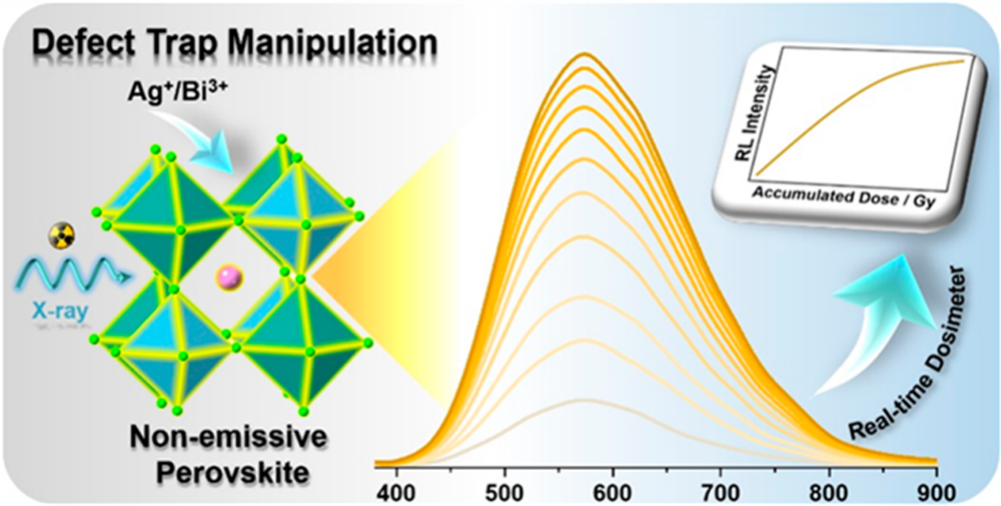

Storage phosphors displaying defect emissions are indispensable in technologically advanced radiation dosimeters. The current dosimeter is limited to the passive detection mode, where ionizing radiation-induced deep-trap defects must be activated by external stimulation such as light or heat. Herein, we designed a new type of shallow-trap storage phosphor by controlling the dopant amounts of Ag^+^ and Bi^3+^ in the host lattice of Cs_2_NaInCl_6_. A distinct phenomenon of X-ray-induced emission (XIE) is observed for the first time in an intrinsically nonemissive perovskite. The intensity of XIE exhibits a quantitative relationship with the accumulated dose, enabling a real-time radiation dosimeter. Thermoluminescence and in situ X-ray photoelectron spectroscopy verify that the emission originates from the radiative recombination of electrons and holes associated with X-ray-induced traps. Theoretical calculations reveal the evolution process of Cl–Cl dimers serving as hole trap states. Analysis of temperature-dependent radioluminescence spectra provides evidence that the intrinsic electron–phonon interaction in 0.005 Ag^+^@ Cs_2_NaInCl_6_ is significantly reduced under X-ray irradiation. Moreover, 0.025 Bi^3+^@ Cs_2_NaInCl_6_ shows an elevated sensitivity to the accumulated dose with a broad response range from 0.08 to 45.05 Gy. This work discloses defect manipulation in halide double perovskites, giving rise to distinct shallow-trap storage phosphors that bridge traditional deep-trap storage phosphors and scintillators and enabling a brand-new type of material for real-time radiation dosimetry.

## Abstract 2

We designed a new type of shallow-trap storage phosphor by controlling the dopant amounts of Ag^+^ and Bi^3+^ in the host lattice of Cs_2_NaInCl_6_. A distinct phenomenon of X-ray-induced emission (XIE) is observed for the first time in these intrinsically nonemissive perovskites. The intensity of XIE exhibits a quantitative relationship with the accumulated dose, enabling a real-time radiation dosimeter.

## Introduction

Storage phosphor is a type of luminescent material that has a function to record the radiation dose.^1−4^ This type of material is extensively utilized for monitoring the accumulated radiation dose in advanced radiation protection technologies, such as personal, medical, space, and security dosimetry.^5−8^ In contrast to the scintillator, radiation-induced charge carriers are stored at defect states in the storage phosphor, forming metastable trapping centers.^4^ The radiation dose is generally proportional to the concentrations of trapped electrons and holes. Then, the trapped electrons and holes would be released through fluorescence emission under external optical/thermal stimulation, providing information on the recorded dose.^2^ In most storage phosphors, oxygen and halogens are commonly used as chemical components, because they can easily form various defects under irradiation. In addition, lanthanide dopants are widely used as trapping centers by adjusting the electronic structure of the host lattice. These material design criteria have created many dosimeter materials in the past several decades, i.e., Al_2_O_3_:C, CaF_2_, CaSO_4_:Dy, and CaS:(Eu, Dy).^9−12^ However, one of the most significant unsolved issues is that the current dosimetry materials cannot assess radiation risk to individuals in real time. From the viewpoint of material design, controlling and manipulating defects are vital and promising for the design of novel real-time radiation dosimeters.

Halide perovskites have recently received considerable attention due to their superior performance in solar cells,^13,14^ light-emitting diodes,^15,16^ photodetectors,^17,18^ etc. In addition, combined with intrinsically high atomic constituents and decent optoelectronic properties, these emergent materials are recognized as promising candidates for next generation radiation detectors.^19−21^ In the past few years, halide perovskites have found substantial utilization in all fields of radiation detection spanning from X-ray imaging and gamma energy spectrum resolution to β-particle detection.^22−25^ The current materials encompass both scintillators and semiconductors; however, they are rarely studied as storage phosphors. Halide perovskites have been proven to have outstanding advantages in the design of storage phosphors. Intrinsic defects, including neutral (*I*_V_), anionic (*I*_V_^+1^), and cationic (*I*_V_^*–*1^) iodine vacancies, are commonly observed in halide perovskites.^26^ Furthermore, the high structural tolerance of the perovskite provides a prerequisite for creating trapping states by doping additional activators, which shows great potential for developing novel radiation dosimeters.

In this work, for the first time, we designed a new type of shallow-trap storage phosphor by controlling the dopant amounts of Ag^+^ and Bi^3+^ in the host lattice of Cs_2_NaInCl_6_. Remarkably, unlike traditional storage phosphors and scintillators, these materials are intrinsically nonemissive under UV excitation but show continuously enhanced emission only under X-ray irradiation, which we define as X-ray-induced emission (XIE). In addition, the emission intensities of 0.005 Ag^+^@ Cs_2_NaInCl_6_ under X-ray irradiation exhibit a simultaneous quantitative relationship with the accumulated dose, enabling a new type of real-time radiation dosimeter. Thermoluminescence (TL) and in situ X-ray photoelectron spectroscopy (XPS) studies demonstrated that this type of emission originates from the radiative recombination of trapped electrons and holes within the X-ray-induced trapping centers. Furthermore, the optimized compound of 0.025 Bi^3+^@ Cs_2_NaInCl_6_ shows a high detection sensitivity to accumulated dose with a broad response range from 0.08 to 45.05 Gy.

## Results and Discussion

### Synthesis, Structure, and X-ray-Induced Emissive Properties of Ag^+^-Doped Cs_2_NaInCl_6_

**Figure 1:**
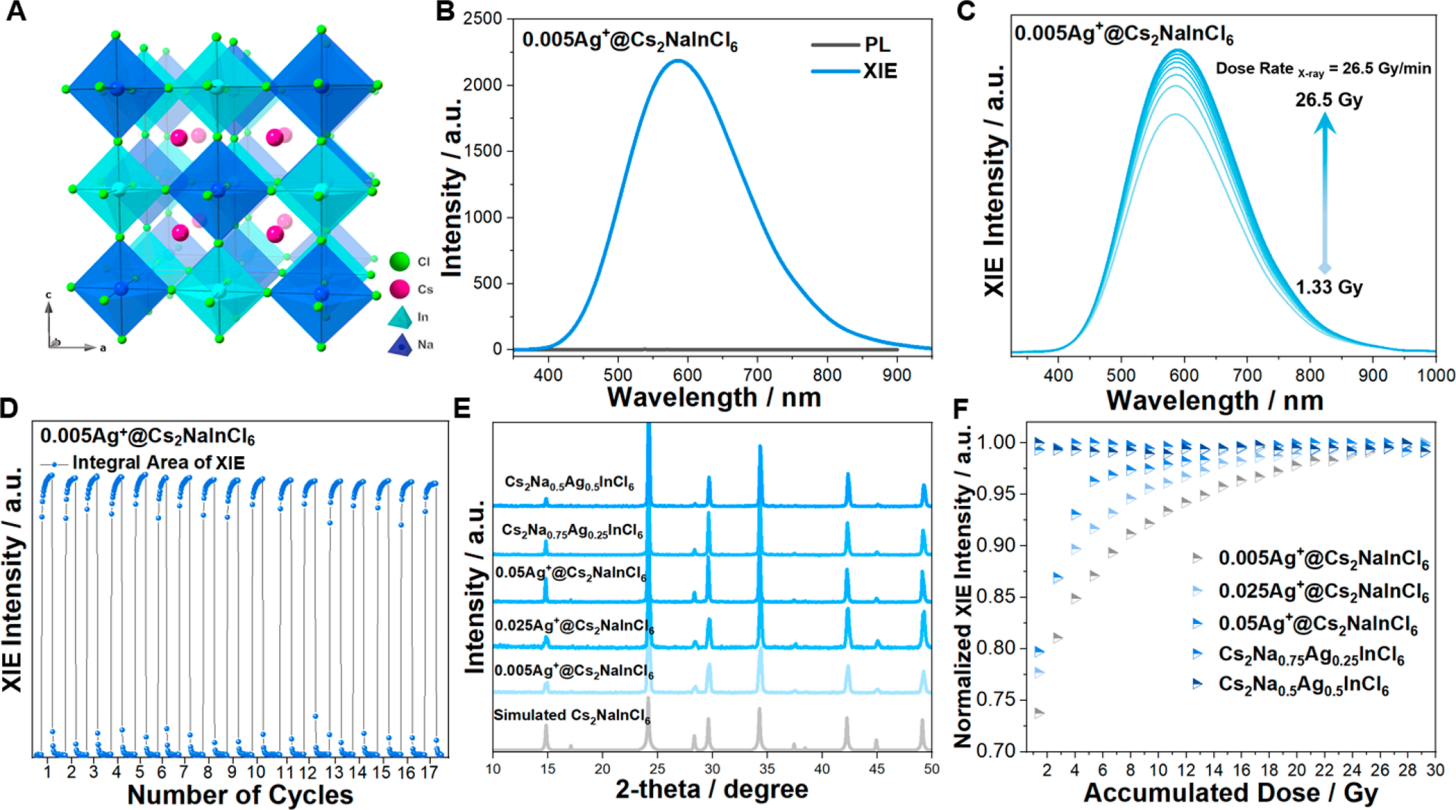
(A) Crystal structure of Cs_2_NaInCl_6_. (B) Comparison of photoluminescence (PL) and XEL spectra at 293 K for 0.005 Ag^+^@ Cs_2_NaInCl_6_. (C) The evolution of XIE spectra of 0.005 Ag^+^@ Cs_2_NaInCl_6_ at a dose rate of 26.5 Gy/min. (D) Cycling performance of 0.005 Ag^+^@ Cs_2_NaInCl_6_ under X-ray irradiation. (E) PXRD patterns of Cs_2_NaInCl_6_ with different Ag^+^-doping ratios. (F) Normalized enhancement processes of XIE intensity with dose accumulation for different Ag^+^-doped Cs_2_NaInCl_6_ samples.

The crystals of Cs_2_NaInCl_6_ and Ag^+^-doped compounds are synthesized under hydrothermal methods through the combination of CsCl, NaCl, InCl_3_, and AgCl with concentrated hydrochloric acid (detailed synthetic procedures can be found in the Supporting Information). Single crystal X-ray diffraction analysis demonstrates that Cs_2_NaInCl_6_ crystallizes in the *Fm*3̅*m* space group with an elpasolite lattice.^27^Figure 1A shows that Cs_2_NaInCl_6_ displays a typical double perovskite structure where the dense three-dimensional structure is formed by corner-sharing [NaCl_6_]^5–^ and [InCl_6_]^3–^ octahedra, with Cs^+^ residing within the cavities. The pure phase is further confirmed by powder X-ray diffraction (PXRD) analysis (Figure S1). We initially investigated the optical properties of Cs_2_NaInCl_6_ under ultraviolet excitation. The direct electronic transitions from the conduction band minimum (CBM) and valence band maximum (VBM) in Cs_2_NaInCl_6_ are theoretically predicted to be parity-forbidden.^28^ Therefore, the absorption peak from 210 to 260 nm originates from the electronic transition between the CBM and the lower level VBM (Figure S2C).^29^ Featuring this electronic structure, Cs_2_NaInCl_6_ presents an intrinsically nonemissive feature at room temperature, consistent with recent reports in which a dark transition leads to undetectable luminescence.^30−32^ To explore the potential possibility of storage phosphor in halide perovskite, we doped a trace amount of Ag^+^ into Cs_2_NaInCl_6_ (named 0.005 Ag^+^@ Cs_2_NaInCl_6_, where 0.005 is the feeding ratio) since Ag^+^ is often used to adjust trapping centers in other storage phosphor materials and will not change the structural integrity of Cs_2_NaInCl_6_.^33,34^ Similar to pure Cs_2_NaInCl_6_, 0.005 Ag^+^@ Cs_2_NaInCl_6_ maintains a nonemissive feature under UV excitation. Intriguingly, under X-ray excitation, it shows a broad emission with a full width at half-maximum of ∼0.7 eV, in sharp contrast to its nonemissive feature when excited by UV irradiation (Figure 1B). Since the nonemissive feature of pure Cs_2_NaInCl_6_ is governed by strong electron–phonon interactions, which has been demonstrated in recent works,^30,31^ this unexpected X-ray-induced emission in a nonemissive perovskite may be attributed to high-energy X-ray irradiation significantly altering the lattice vibrations of Ag^+^-doped Cs_2_NaInCl_6_, potentially affecting the electron–phonon interactions. To demonstrate this hypothesis, we examined the Huang–Rhys factor (*S*) of 0.005 Ag^+^@ Cs_2_NaInCl_6_ under X-ray irradiation. *S* quantifies the number of phonons emitted with excited state relaxation after photoexcitation^35−37^ and is expressed as *S* = Δ*E*/ℏω, where Δ*E* is the relaxation energy of the excited states and ω is the frequency of the longitudinal optical (LO) phonon.^35,38^ Recent theoretical studies have indicated that Cs_2_NaInCl_6_ has an intrinsically strong Huang–Rhys factor with a value larger than 80. Interestingly, under X-ray excitation, we observed a significant reduction in the Huang–Rhys factor, with a calculated value of 11.66 (Figure S3A and B), suggesting a decrease in electron–phonon interactions. This observation demonstrates that X-rays induce a new relaxation process of excited states, resulting in a lattice that is more prone to deformation under X-ray irradiation, presenting a higher radiative transition probability, as shown in Figure S3C.

More remarkably, the emission intensity of the 0.005 Ag^+^@ Cs_2_NaInCl_6_ single crystal significantly increases as the X-ray irradiation dose increases, contrasting sharply with traditional scintillators that emit at a constant intensity. We collected the emission spectrum with gradient radiation dosages and found that the emission intensity reached saturation after exposure to a radiation dose of 26.5 Gy (Figure 1C). Furthermore, the emission of 0.005 Ag^+^@ Cs_2_NaInCl_6_ exhibits a nonlinear dependence on the accumulated dose, with the luminescence intensity sharply increasing under initial X-ray irradiation and then reaching saturation with increasing dosage (Figure S2A). In addition, the emission process can be recycled constantly and maintains the stability of intensity (Figure 1D). This XIE feature reveals that 0.005 Ag^+^@ Cs_2_NaInCl_6_ can accumulate the received radiation dosage in real time, and we thus classify this material as a storage phosphor to distinguish it from the traditional scintillator.

We also synthesized other crystals of Cs_2_NaInCl_6_ with higher contents of Ag^+^. The PXRD data show that the different Ag^+^-doping ratio samples sustain the initial Cs_2_NaInCl_6_ structure. Besides, a gradual shift of the characteristic diffraction peaks (2 2 0) to higher angles, approximately at 24°, which suggests that the doped Ag^+^ ions locate within the Na^+^ sites in the Cs_2_NaInCl_6_ crystal structure (Figures 1E and S2B). However, with increased Ag^+^ components, the absorption properties display obvious differences and are consistent with the reported results (Figure S2C).^31,32^ Correspondingly, the emission properties are also different. For 0.025 and 0.05 Ag^+^@ Cs_2_NaInCl_6_, they are still nonemissive under UV excitation but possess luminescent processes similar to those of 0.005 Ag^+^@ Cs_2_NaInCl_6_ under X-ray irradiation; the difference is the enhancement processes of emission intensity versus dose accumulation (Figure 1F). In contrast, Cs_2_Na_0.25_Ag_0.75_InCl_6_ and Cs_2_Na_0.5_Ag_0.5_InCl_6_ are emissive under both UV and X-ray excitation (Figure 1F and Table 1), which is attributed to the breakthrough of the parity-forbidden transition after large amounts of Ag^+^ doping and is consistent with the reported results.^31^ The sustained emission intensity of these materials under X-ray irradiation indicates that they can be categorized as scintillators as opposed to storage phosphors.

**Table 1:**
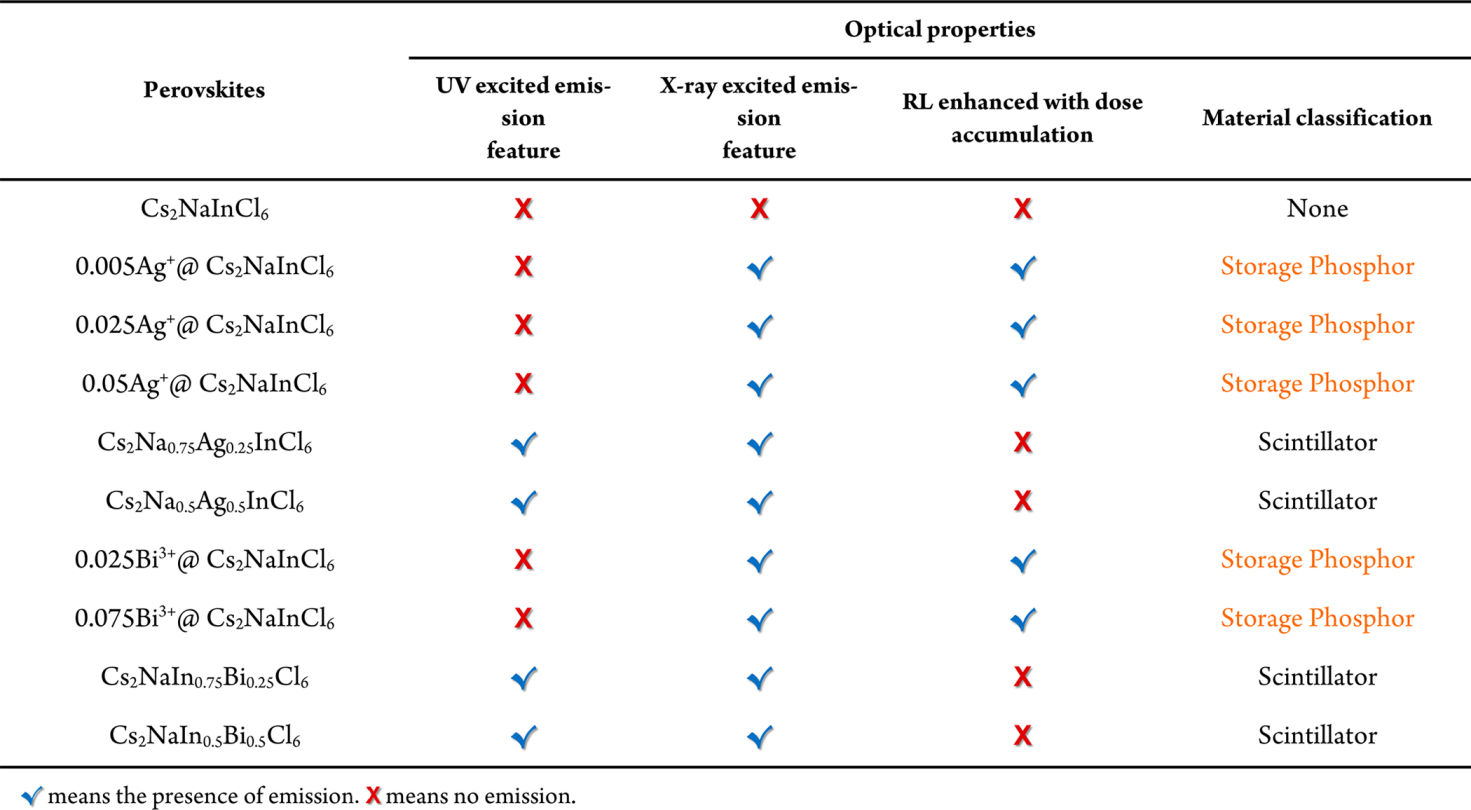
Summary of the Optical Properties of Cs_2_NaInCl_6_ with Different Ag^+^ and Bi^3+^ Ion Ratios

### The X-ray-Induced Trap Formation Mechanism

**Figure 2:**
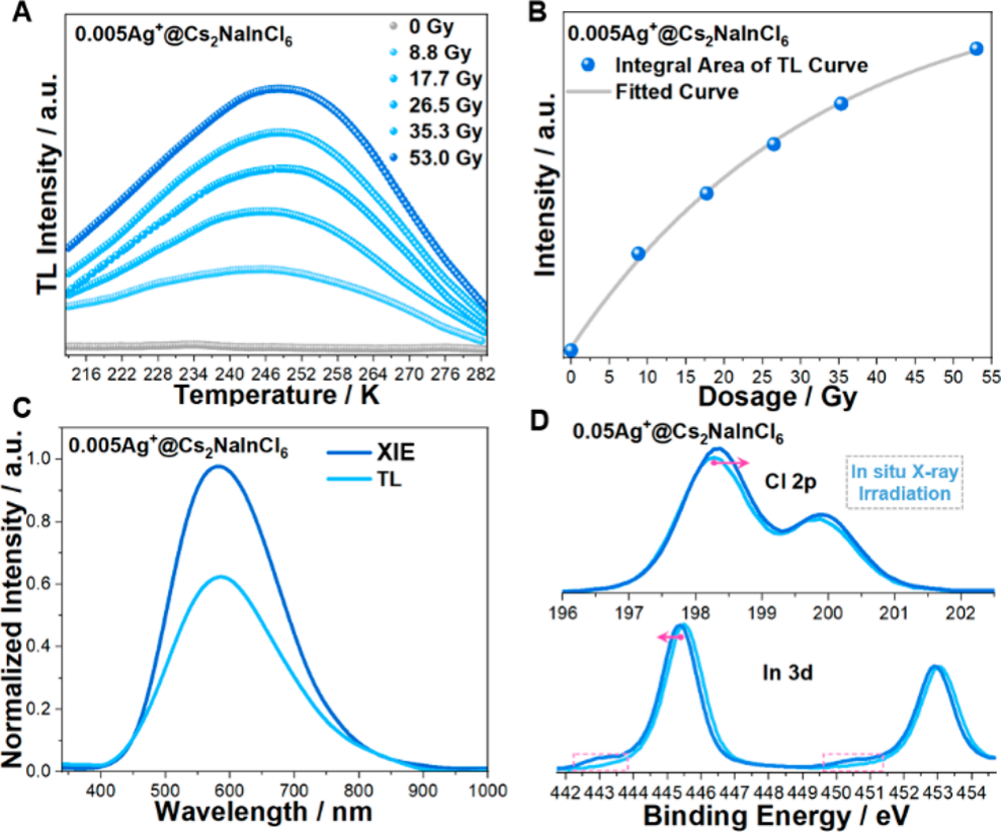
(A) Thermoluminescence (TL) curves of 0.005 Ag^+^@ Cs_2_NaInCl_6_ under incremental X-ray dosages from 213 to 285 K. (B) The integral area of TL curves versus received dosage. (C) Comparison of the XIE and TL spectra. (D) In situ measured XPS (Al Kα) of 0.05 Ag^+^@ Cs_2_NaInCl_6_ under continuous X-ray irradiation. The pink arrow indicates a peak shift, and the dotted line frame indicates a new peak.

Considering the proper emissive character of Ag^+^-doped Cs_2_NaInCl_6_, it is suspected that the luminescence may originate from trap recombination induced by X-ray irradiation.^39,40^ To understand the trap property of Ag^+^-doped Cs_2_NaInCl_6_, we measured the thermoluminescence (TL) spectrum of 0.005 Ag^+^@ Cs_2_NaInCl_6_ under X-ray irradiation. TL measurement is commonly used to verify the existence of traps that are generated by irradiation in storage phosphor materials.^41^ The crystals were initially frozen at 213 K and then irradiated with X-rays at various dosages. After the X-ray source was turned off, the crystals showed a significant afterglow at 213 K compared to that at room temperature, indicating the presence of traps. Additionally, the afterglow time increased with increasing X-ray dose (Figure S4 and Table S1). When the afterglow spectrum was almost undetectable at 213 K, TL spectra were collected at increasing temperatures with a ramp-up rate of 0.17 °C/s. As shown in Figure 2A, compared with the case with no X-ray irradiation, significant TL phenomena are observed under incremental irradiation dosages, demonstrating that X-rays create substantial traps within 0.005 Ag^+^@ Cs_2_NaInCl_6_. Furthermore, a broad band located at 248.5 K was recorded in the TL curves, implying that X-rays solely generate one type of trap state in 0.005 Ag^+^@ Cs_2_NaInCl_6_.^42^ In addition, Figure 2B shows the integral area of the TL curve dependency on the received dosage, indicating that the trap concentration is highly correlated to the irradiated dosage. The above results directly illustrate that a new storage phosphor has been designed in the halide double perovskite 0.005 Ag^+^@ Cs_2_NaInCl_6_. Based on the TL curves, the trap depth in 0.005 Ag^+^@ Cs_2_NaInCl_6_ was calculated using the peak shape method (detailed calculations are shown in Methods).^43,44^ A shallow trap depth of ∼0.30 eV was obtained, which is different from traditional storage phosphor materials that usually have deep trap depths (higher than 0.6 eV, as shown in Table S2) requiring external optical/thermal stimulation to release the traps.^9−11^ This shallow trap depth is inferred to make trapped electrons and holes spontaneously released at room temperature, leading to the dosage accumulation effect. In addition, the peak shape and position of the TL are identical with those of XIE, confirming that the XIE of 0.005 Ag^+^@ Cs_2_NaInCl_6_ predominantly originates from the recombination of the same electron and hole traps (Figure 2C).

Different from UV light, X-rays have enough energy to create various point defects in bulk materials through ionization interactions, which can serve as electron or hole traps. According to the established theory of X-ray-matter interactions, Cl^–^ will be dislocated and form a well-known *V*_*k*_ center (Cl_2_^–^) with another adjacent Cl^–^.^4,45^ Cl_2_^–^ is a typical hole trapping center in halide perovskites and is believed to play a critical role in Ag^+^-doped Cs_2_NaInCl_6_.^41,46^ To explore the intrinsic trapping process of excited electrons and holes, we investigated in situ XPS to reveal the X-ray-induced electron transfer behavior. Under continuous X-ray irradiation, the Cl 2p peaks in both 0.05 Ag^+^@ Cs_2_NaInCl_6_ and pure Cs_2_NaInCl_6_ notably shift to higher binding energies, indicating that electron-losing behavior appears in the Cl atom and the induction of hole-trapping centers (Cl_2_^–^) by X-ray excitation (Figures 2D and S5C). However, the behavior of In 3d in 0.05 Ag^+^@ Cs_2_NaInCl_6_ is totally different from that in pure Cs_2_NaInCl_6_, with a shift to lower binding energy and the appearance of two new shoulder peaks in In 3d_3_ and 3d_5_ at lower binding energy, indicating the electron-withdrawing character of In in Ag^+^-doped Cs_2_NaInCl_6_ under X-ray irradiation (Figures 2D and S5A). In contrast, there is no obvious change in In 3d in pure Cs_2_NaInCl_6_ (Figure S5D). Additionally, the microcomponent of Ag 3d also shows a detectable shift to a lower binding energy in in situ XPS after irradiation (Figure S5B). These hints suggest that Ag^+^ doping promotes the formation of electron trapping centers in halide double perovskite Cs_2_NaInCl_6_ under X-ray irradiation.

**Figure 3:**
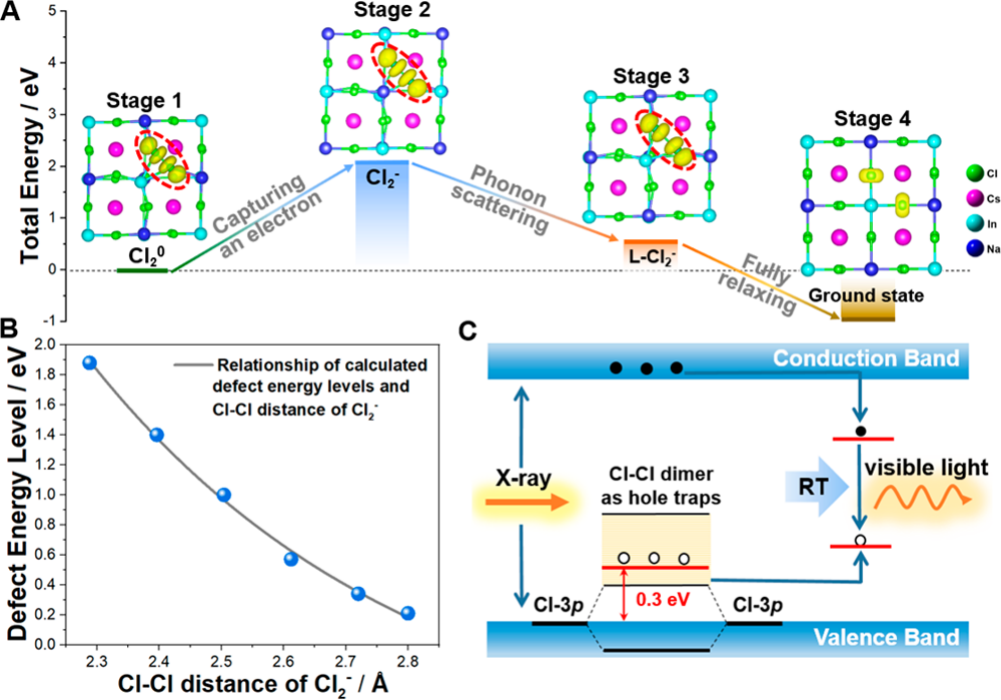
(A) Evolution process of destruction of the Cl–Cl dimer in Cs_2_NaInCl_6_. (B) Relationship of calculated trap energy levels and the Cl–Cl distance of Cl_2_^–^. (C) Schematic diagram of defect formation and the combination process in 0.005 Ag^+^@ Cs_2_NaInCl_6_ under X-ray irradiation.

Moreover, density functional theory calculations were carried out to clarify the role of the Cl_2_^–^ hole trapping centers induced by X-rays in Cs_2_NaInCl_6_ (detailed calculation methods can be found in the Methods). The calculation showed that two adjacent Cl atoms form a stable Cl_2_^0^ dimer structure, with the Cl–Cl distance decreasing from 3.63 to 2.01 Å (Stage 1 in Figure 3A). The trap state (red line in Figure S6A) appears in the band gap and serves as a hole trap mainly derived from the Cl–Cl antibonding orbitals. According to experimental observations, the traps can be released at room temperature, which is probably due to the destruction of the Cl_2_^0^ dimer after trapping electrons. Therefore, an electron was introduced into Cl_2_^0^ to simulate the stability of the Cl_2_^–^ dimer structure. As shown in Figure 3A, the level of the Cl_2_^–^ dimer (Stage 2) is 2.10 eV higher than that of the Cl_2_^0^ dimer in total energy, implying that the Cl_2_^–^ dimer can spontaneously recover and relax into the low-lying energy state by phonon scattering (Stage 3, defined as L-Cl_2_^–^). Finally, L-Cl_2_^–^ fully relaxes to the initial state of Cs_2_NaInCl_6_ by capturing an electron (Stage 4). The relaxation of the Cl_2_^–^ dimer is in a broad energy range due to multiple lattice vibrations, and the energy level of traps gradually decreased from 1.88 to 0.21 eV, accompanied by Cl–Cl distance relaxation to equilibrium status (Figures 3B and S6). The experimental value of 0.30 eV from the TL measurements is right in the range, corroborating the defects’ origin under X-ray excitation. Overall, the in situ XPS and calculation results indicate that Cl_2_^–^ serves as a hole trap to capture holes under X-ray excitation in the storage phosphor perovskite. At the same time, the trace doping ratio of Ag^+^ promotes the trapping of electrons in the In^3+^ site, and the spontaneous radiative recombination of trapped electrons and holes leads to the XIE (Figure 3C).

**Figure 4:**
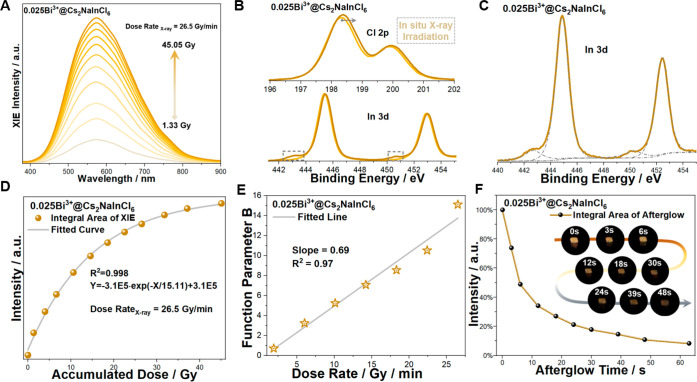
(A) The evolution of the XIE spectra of 0.025 Bi^3+^@ Cs_2_NaInCl_6_ at a dose rate of 26.5 Gy/min. (B) In situ measured XPS (Al Kα) of 0.025 Bi^3+^@ Cs_2_NaInCl_6_ under continuous X-ray irradiation. The gray arrow indicates a peak shift, and the dotted line frame indicates a new peak. (C) Peak-differentiation and imitating of new peaks in In 3d XPS data after continuous X-ray irradiation. (D) The XIE intensity of 0.025 Bi^3+^@ Cs_2_NaInCl_6_ as a function of accumulated dosage. (E) Fitted function parameter B with received dose rate. (F) The afterglow for 0.025 Bi^3+^@ Cs_2_NaInCl_6_ with X-ray cutoff. The inset illustrates the afterglow intensity of a single crystal over the collection time.

### Regulating Trap Characteristics of Cs_2_NaInCl_6_ for Improving Detection Sensitivity

The above results provide a new type of storage phosphor in Cs_2_NaInCl_6_ with a trace doping ratio of Ag^+^, and the continuously increased emission, depending on the accumulated X-ray dose, illustrates intrinsic real-time dosimetry. However, 0.005 Ag^+^@ Cs_2_NaInCl_6_ only works in a narrow dosage region, as shown in Figure 1C. Since the X-ray-induced emission is associated with defect trap state formation, while the In^3+^ site is the electron trapping center, we proposed that doping guest ions on this site would regulate the trap characteristics of Cs_2_NaInCl_6_. We thus incorporate Bi^3+^ ions into the lattice that would sustain the structural integration of Cs_2_NaInCl_6_.^27^ Here, we name the samples 0.025 Bi^3+^@ Cs_2_NaInCl_6_, 0.075 Bi^3+^@ Cs_2_NaInCl_6_, Cs_2_NaIn_0.75_Bi_0.25_Cl_6_, and Cs_2_NaIn_0.5_Bi_0.5_Cl_6_ based on different Bi^3+^ feeding ratios. The accurate amounts of Bi^3+^ and In^3+^ were confirmed by ICP–OES, as shown in Table S3, and the results are consistent with the increased Bi^3+^ feeding ratio. Clearly, four different samples maintain the initial structure (Figure S7) but exhibit distinctively different lattice vibrations (Figure S8). We investigated the optical properties of these samples under UV and X-ray irradiation. Similar to the Ag^+^-doped samples, only 0.025 Bi^3+^@ Cs_2_NaInCl_6_ and 0.075 Bi^3+^@ Cs_2_NaInCl_6_ with trace amounts of Bi^3+^ doping exhibit storage phosphor characteristics, as shown in Figures 4A and S9. In contrast, the samples of Cs_2_NaIn_0.75_Bi_0.25_Cl_6_ and Cs_2_NaIn_0.5_Bi_0.5_Cl_6_ show distinct optical properties, manifesting direct UV excited emission and scintillating phenomena (Table 1 and Figures S9). The electronic transition spectra in Figure S10 indicate that a high doping ratio results in efficient electronic transfer between the [BiCl_6_]^3–^ group and the [InCl_6_]^3–^ group and thus changes the electronic structure of Cs_2_NaIn_0.75_Bi_0.25_Cl_6_ and Cs_2_NaIn_0.5_Bi_0.5_Cl_6_. Intriguingly, unlike 0.005 Ag^+^@ Cs_2_NaInCl_6_, 0.025 Bi^3+^@ Cs_2_NaInCl_6_ has decent detection sensitivity with a dosage response range from 0.08 to 45.05 Gy (Figure 4A and Figure S11), which substantially covers technical demands in radiation detection fields. The in situ XPS analysis illustrates a similar trapping process of excited electrons and holes with 0.05 Ag^+^@ Cs_2_NaInCl_6_, as shown in Figure 4B and C. In addition, the electron-losing behavior in Bi 4f_7_ shows that Bi may be involved in the hole-trapping process (Figure S12). We also measured the TL curves of 0.025 Bi^3+^@ Cs_2_NaInCl_6_ and calculated the trap depth as ∼0.40 eV, which is higher than that of 0.005 Ag^+^@ Cs_2_NaInCl_6_ (Table S1 and Figure S13). By fitting the data in Figure 4A, we can quantitatively evaluate the emission intensity as a function of radiation dose from 1.33 to 45.05 Gy (Figure 4D). The equation *Y* = *A*·exp(−*X*/*B*) + *C* is then deduced, where *A* and *C* are constant parameters and *B* is a parameter associated with the dosage rate. Figure 4E shows the relationship between parameter B and the dosage rate. A linear dependence was obtained, indicating that 0.025 Bi^3+^@ Cs_2_NaInCl_6_ has the intrinsic property of dosage accumulation, regardless of the variation in the dose rate. The above dose-dependent relationship suggests the application potential in real-time visualized radiation dosimetry. Furthermore, 0.025 Bi^3+^@ Cs_2_NaInCl_6_ exhibits a relatively long afterglow of more than 63 s (Figure 4F), making it promising for applications such as radiation medical imaging.^47^ In addition, the dynamic process of enhanced emission intensity and long afterglow is also recorded in the Supplementary Video.

## Conclusions

The XIE phenomenon was observed for the first time in the shallow-trap storage phosphors reported in this work. These materials are not emissive under UV excitation, but they show a continuously enhanced emission under X-ray irradiation. Traditional storage phosphors, such as Al_2_O_3_:C, CaF_2_, and CaSO_4_:Dy, widely used for dosimetry, are based on deep-trap materials significantly deviating from the XIE. In practice, these materials cannot work in real-time mode and require additional external stimulations (generally, thermal stimulation at 575 K in a specialized instrument). Traditional scintillators display emission that can be excited by both UV and X-ray irradiation with the emission intensity remaining constant under continuous X-ray irradiation, thus excluding the XIE phenomenon. Importantly, the XIE property is directly related to the doping ratio of Ag^+^ and Bi^3+^ in the host lattice Cs_2_NaInCl_6_. As demonstrated in Table 1, the materials with trace amounts of doping ions display an XIE phenomenon but with no emission feature under UV excitation. This class of materials can be classified as shallow-trap storage phosphors. In contrast, the electronic structure of high dopant materials is distinct from that of low dopant materials. When the ratio of Na:Ag and In:Bi is higher than 3 (molar ratio is 0.75:0.25), these materials display a scintillation phenomenon, with a sustained emission intensity under X-ray excitation as well as emission under UV excitation. The proposed doping strategy provides a distinct advantage in achieving the XIE phenomenon, which distinguishes shallow-trap storage phosphors from traditional X-ray-sensitive materials and enables the development of a new type of material suitable for real-time radiation dosimetry.

In conclusion, we disclose a new type of storage phosphor in nonemissive halide double perovskite Cs_2_NaInCl_6_ by combining defect traps, which enables the development of real-time radiation dosimeters. Thermoluminescence and in situ XPS measurements and theoretical calculations indicate that trap formation and radiative recombination under X-ray irradiation result in this phenomenon. The depth of the shallow trap facilitates the spontaneous recombination of trapped electrons and holes at room temperature, leading to a dosage accumulation effect. In addition, we observe that the intrinsic electron–phonon interaction in 0.005 Ag^+^@ Cs_2_NaInCl_6_ is significantly reduced under X-ray irradiation. Finally, by regulating the trap characteristics of Cs_2_NaInCl_6_, we demonstrate that 0.025 Bi^3+^@ Cs_2_NaInCl_6_ has a decent detection sensitivity with a dosage response range from 0.08 to 45.05 Gy, which substantially covers technical demands in radiation monitoring fields. These findings not only provide a new horizon into the structure–property relationship of solids under irradiation with high-energy photons but also disclose a new attractive application for perovskite-based materials.

## Supporting Information Available

Experimental procedures; materials and computational methods, characterization data indculding XRD, XIE spectra, XPS data, Raman spectra, band structure results (Figures S1–S14), and supplementary tables (Tables S1–S4) (PDF)
Movie S1: Dynamic process of X-ray-induced emission and afterglow of 0.025 Bi^3+^@ Cs_2_NaInCl_6_ (MP4)
