# Supplementary material for: Manipulation of Shallow-Trap States in Halide Double Perovskite Enables Real-Time Radiation Dosimetry
Source: ACS Cent Sci. 2023 Sep 11;9(9):1827–34. doi: 10.1021/acscentsci.3c00691 (PMC10540297; doi:10.1021/acscentsci.3c00691)
Supplement: Supplementary file 1 — oc3c00691_si_001.pdf [file oc3c00691_si_001.pdf]

Supporting Information

Manipulation of Shallow-Trap States in Halide Double Perovskite Enables Real-time Radiation Dosimetry

Yumin Wang,[1,†] Gaoyuan Chen,[3,2,†] Zibin Zhu,[1] Haoming Qin,[1] Liangwei Yang,[1] Duo Zhang,[1] Yingguo Yang,[4] Menglin Qiu,[5] Ke Liu,[4] Zhifang Chai,[1] Wanjian Yin,[2,*] Yaxing Wang,[1,*] and Shuao Wang[1,*]

[1] State Key Laboratory of Radiation Medicine and Protection, School for Radiological and Interdisciplinary Sciences (RAD-X) and Collaborative Innovation Center of Radiation Medicine of Jiangsu Higher Education Institutions, Soochow University, Suzhou 215123, China.

[2] College of Energy, Soochow Institute for Energy and Materials Innovations (SIEMIS), Jiangsu Provincial Key Laboratory for Advanced Carbon Materials and Wearable Energy Technologies, Soochow University, Suzhou 215006, China.

[3] Jiangsu Key Laboratory of Micro and Nano Heat Fluid Flow Technology and Energy Application, School of Physical Science and Technology, Suzhou University of Science and Technology, Suzhou, 215009, China

[4] Shanghai Synchrotron Radiation Facility (SSRF), Zhangjiang Lab, Shanghai Advanced Research Institute, Shanghai Institute of Applied Physics, Chinese Academy of Sciences, Shanghai 201204, China.

[5] Key Laboratory of Beam Technology of Ministry of Education, College of Nuclear Science and Technology, Beijing Normal University, Beijing 100875, China.

Materials and Methods

Materials.

CsCl (99.999%, Strem Chemicals, Inc), NaCl (99.999%, Aladdin), InCl (99.995%, ThermoFisher Scientific), AgCl (99.5%, Aladdin), $Bi(NO_3)_3 \cdot 5H_2O$ (99.999%, ThermoFisher Scientific), and HCl (AR, 36~38%, Sinopharm Chemistry Reagent Co., Ltd) were used as received from commercial suppliers without further purification.

Synthesis.

CsCl (0.4 mmol), 0.2 mmol NaCl and 0.2 mmol InCl3 were mixed with 2 mL 12 M HCl and added into a 23 mL PTEF-lined autoclave to synthesize the pure phase of $Cs_2NaInCl_6$. AgCl (0.001 mmol, 0.005 mmol and 0.01 mmol) was added to the mixture for the synthesis of $0.005Ag^+@Cs_2NaInCl_6$, $0.025Ag^+@Cs_2NaInCl_6$ and $0.05Ag^+@Cs_2NaInCl_6$, respectively. In addition, $Cs_2Na_{0.75}Ag_{0.25}InCl_6$ and $Cs_2Na_{0.5}Ag_{0.5}InCl_6$ were synthesized by adding x mmol NaCl and 0.2-x mmol AgCl (x= 0.15, 0.1). The synthesis of Bi3+-doped samples is similar to that of Ag^+-doped samples, except for choosing $Bi(NO_3)_3 \cdot 5H_2O$ as the raw material for Bi^{3+}. All the mixtures were heated at 180 °C for 12 h and cooled to room temperature at a rate of 3 °C/h. Colorless crystals were obtained by washing them with ionized water and isopropanol.

Physical Property Measurements.

Powder X-ray diffraction patterns were collected by using a Bruker D8 Advance X-ray diffractometer with Cu–K_α radiation (λ = 1.54056 Å). Raman spectroscopy, UV–vis absorption and photoluminescence (PL) spectrum data were measured on the single crystals using a Craic Technologies microspectrophotometer. X-ray excited radioluminescence (RL) and afterglow spectra were collected on an X-RAD SmART system equipped with a W-K_α radiation source (beam size of X-ray is 10×10 cm^2) and a NOVA spectrometer (ideaoptics, China) with different dosage rates. The dosage rate was changed by adjusting the tube voltage and electricity. In addition, the accumulated doses were acquired by multiplying the dose rate and cumulative exposure time of the material under X-ray irradiation. The sizes of the single crystals chosen for RL-related measurements are both approximately 1×1×1 mm^3. Temperature-dependent RL and thermoluminescence spectra were detected by coupling a Linkam Temperature Controlled Microscope Stage, as shown in Figure S14. Inductively coupled plasma optical emission spectrometer (ICP–OES) analysis of separation was conducted using a Thermo Scientific ICAP 7400 instrument. X-ray photoelectron spectroscopy (XPS) spectra were collected with a Thermo escalab 250Xi (Al–K_α). For in situ measurements, we collected spectra every four minutes three times with continuous exposure to X-ray irradiation. Moreover, three spectra were collected at the same site.

Computational Method.

Density functional theory calculations with the generalized gradient approximation of Perdew-Burke-Ernzerhof (GGA-PBE)[1] were carried out based on all-electron-like projector-augmented wave (PAW) potentials,[2] as implemented in the Vienna Ab initio Simulation Package (VASP).[3,4] Monkhorst-Pack k-point meshes[5] of 7×7×7 were adopted for a (10.73 Å × 10.73 Å × 10.73 Å) supercell containing 40 atoms, and a plane-wave cut-off energy of 400 eV was used for the self-consistent calculations.

Calculation of the Huang–Rhys factors.

The Huang–Rhys factor (S) can be obtained by the following equation:

$$\mathrm{FWHM} = 2.36\sqrt{S}\hbar\omega_{\mathrm{phonon}}\sqrt{\coth\frac{\hbar\omega_{\mathrm{phonon}}}{2k_{\mathrm{B}}T}}$$

where FWHM is the full width at half maximum of temperature-dependent radioluminescence and $\hbar\omega_{\mathrm{phonon}}$ is the phonon energy.

Calculation of trap depth.

The trap depths can be calculated from the peak shape method based on TL curves. The peak shape method according to following Equation.[6,7]

$E = c_{□} k T_m^2/□ - b_{□}(2kT_m)$,

where E is trap depth, □ is □ (refer to the total half-width of the TL glow peak). k is Boltzmann's constant and Tm is the absolute temperature of the maximum intensity of TL peak. In addition, the value of $c_{□}$ and $b_{□}$ rely on the kinetics which determined by the shape factor $□_g$ ($□_g = □/□$, □ refers to the half-width at the high temperature side). The $□_g$ values for $0.005Ag^{+}@Cs_2NaInCl_6$ and $0.025Bi^{3+}@Cs_2NaInCl_6$ were calculated to be ~0.45 and ~0.46, respectively. These values fall within the range of 0.42-0.52, indicating a typical characteristic of general-order kinetics. Therefore, the values of c□ and b□ were determined to be 2.826 and 1, respectively. This allowed us to calculate the trap depths, which were ~0.30 eV and ~0.40 eV for $0.005Ag^{+}@Cs_2NaInCl_6$ and $0.025Bi^{3+}@Cs_2NaInCl_6$, respectively.

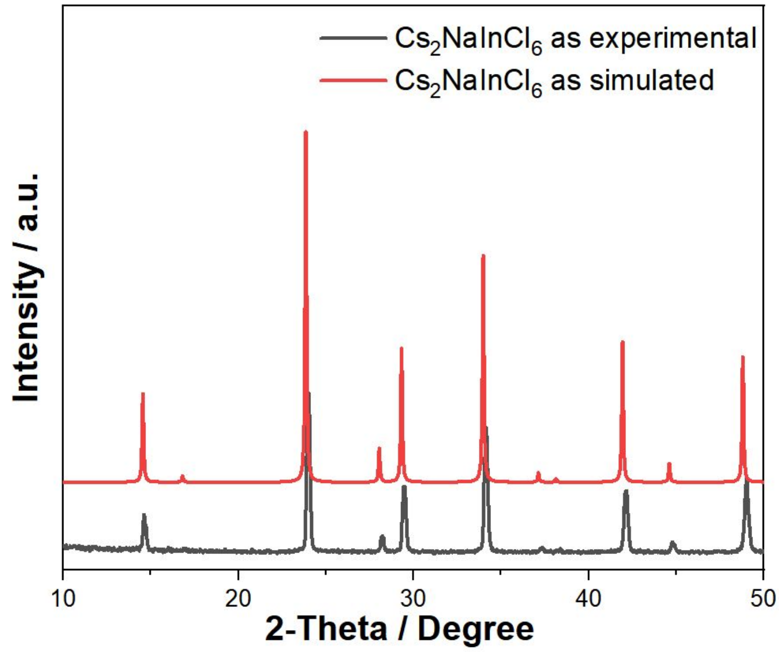

Figure S1. Experimental and simulated X-ray powder diffraction patterns of $Cs_2NaInCl_6$.

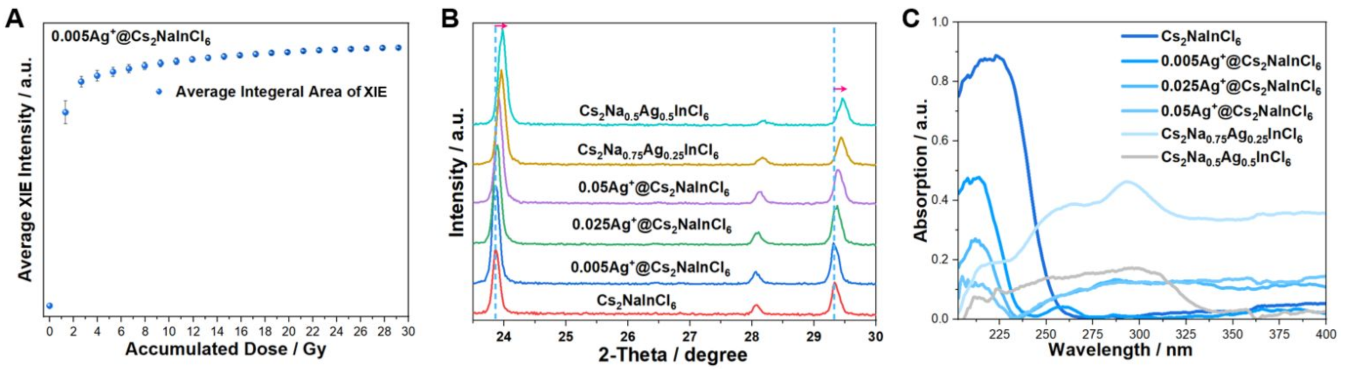

Figure S2. (A) Average XIE intensity versus accumulated X-ray dose of $0.005Ag^+$@ $Cs_2NaInCl_6$. (B) Enlarged PXRD patterns of $Cs_2NaInCl_6$ with different Ag^+-doping ratios; (C)Absorption spectra of $Cs_2NaInCl_6$ with various Ag^+ ion contents.

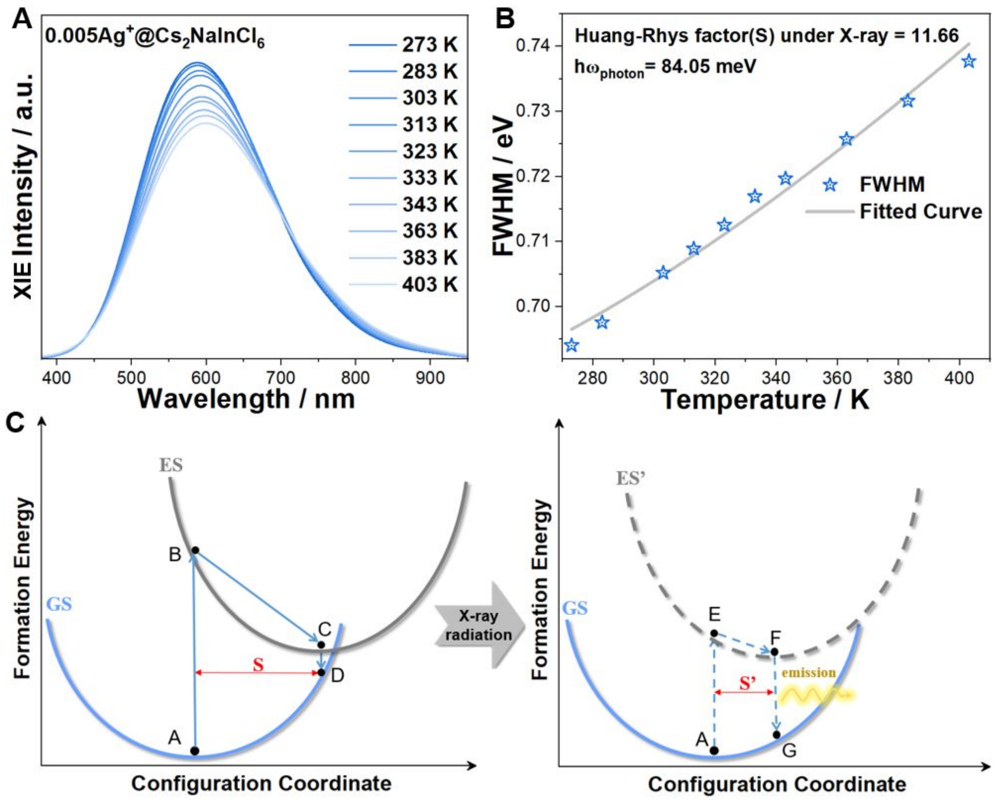

Figure S3. (A) FWHM broadening of radioluminescence spectra at different temperatures of $0.005Ag^{+}@Cs_2NaInCl_6$. (B) Fitted curve for FWHM of radioluminescence spectra with related temperature. (C) Configuration coordinate model describing the interactions between electrons and phonons in $0.005Ag^{+}@Cs_2NaInCl_6$ without (left) and with (right) X-ray irradiation.

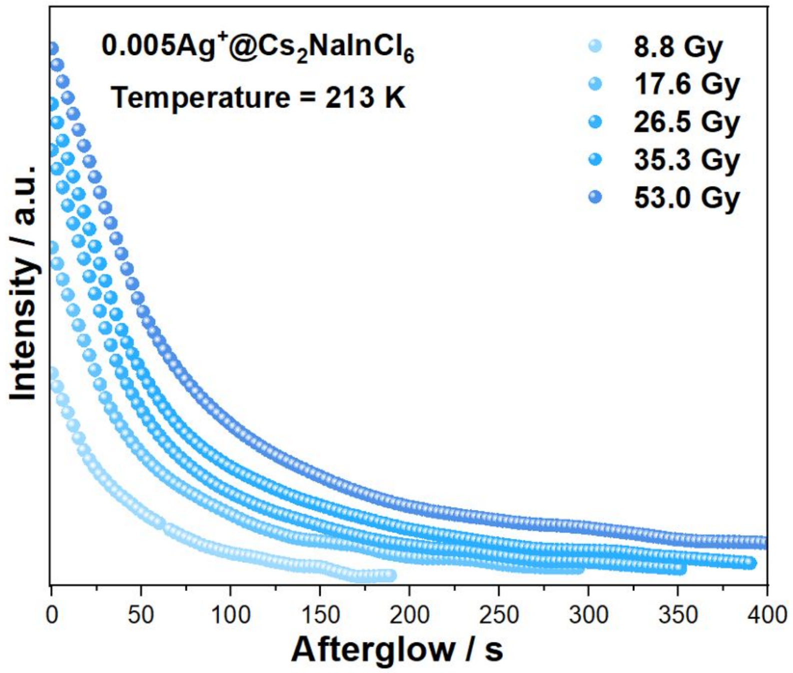

Figure S4. The afterglow of the $0.005Ag^{+}@Cs_2NaInCl_6$ single crystal irradiated with incremental X-ray dosages at 213 K.

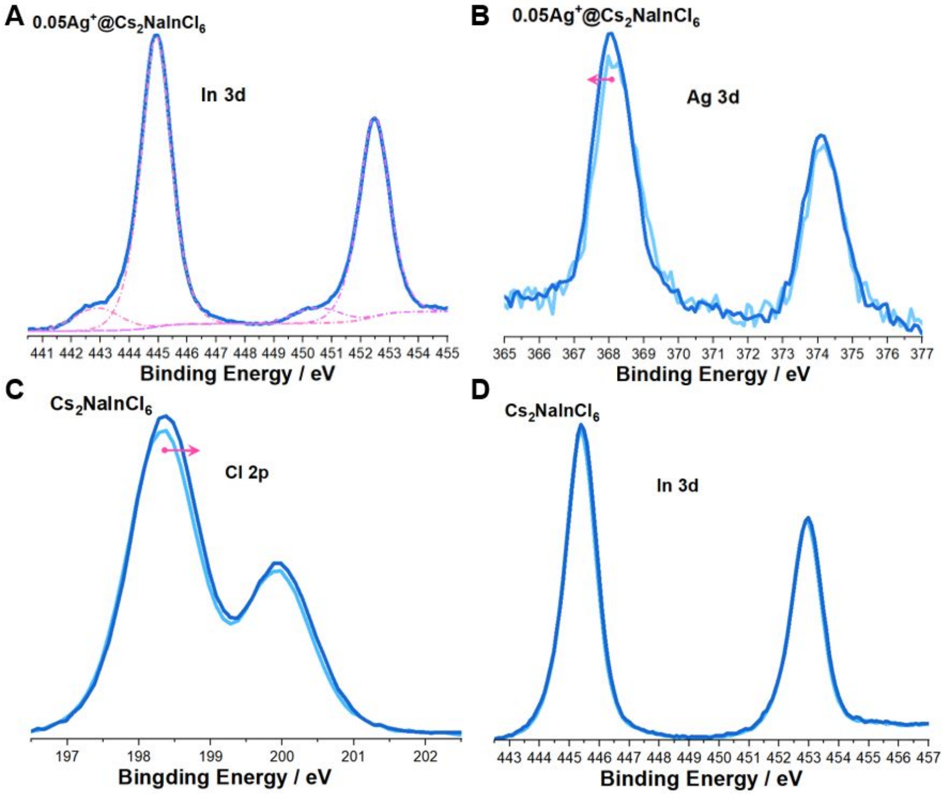

Figure S5. (A) Peak-differentiating and imitating of new peaks in In 3d in situ XPS spectra after continuous X-ray irradiation. (B) The change in Ag 3d in situ XPS spectra of $0.005Ag^{+}@Cs_2NaInCl_6$ under continuous X-ray irradiation. (C, D) Changes in Cl 2p and In 3d in situ XPS spectra of pure $Cs_2NaInCl_6$ under continuous X-ray irradiation.

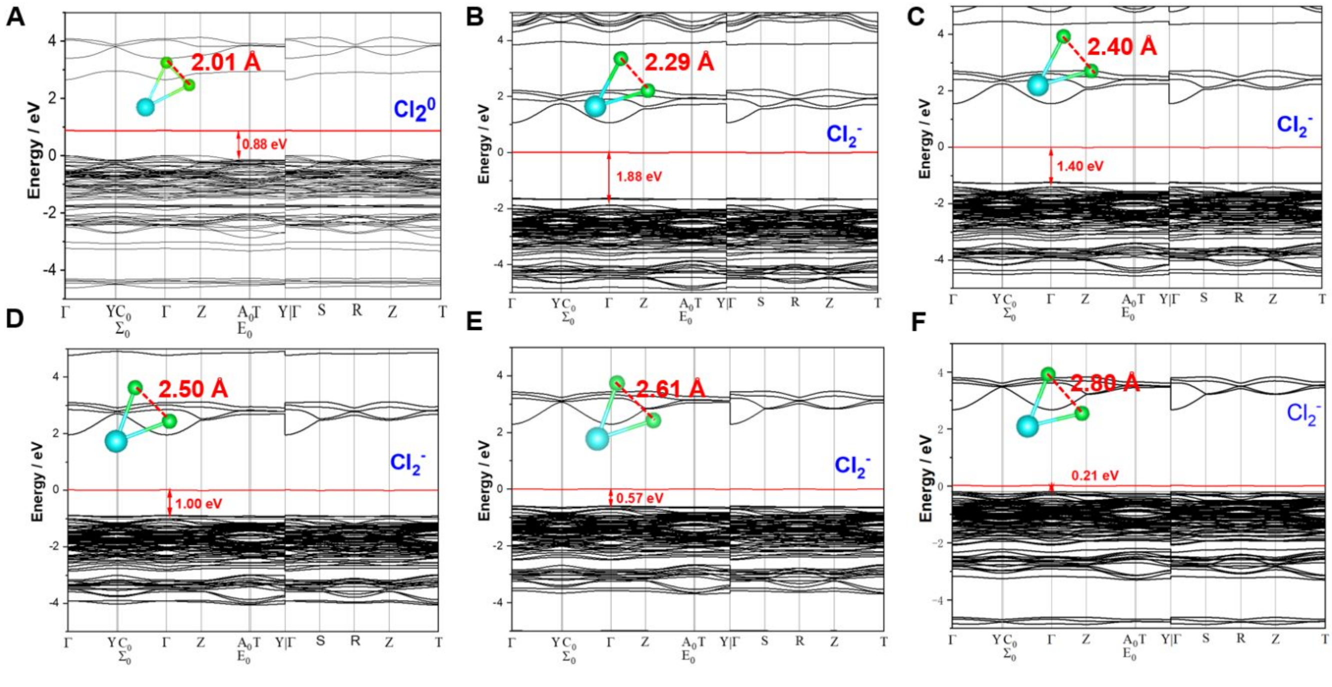

Figure S6. (A) Band structure of Cl_2^0-$Cs_2NaInCl_6$. (B) Band structure of Cl_2^--$Cs_2NaInCl_6$ at 2.29 Å, (C) 2.40 Å, (D) 2.50 Å, (E) 2.61 Å and (F) 2.80 Å Cl-Cl distances.

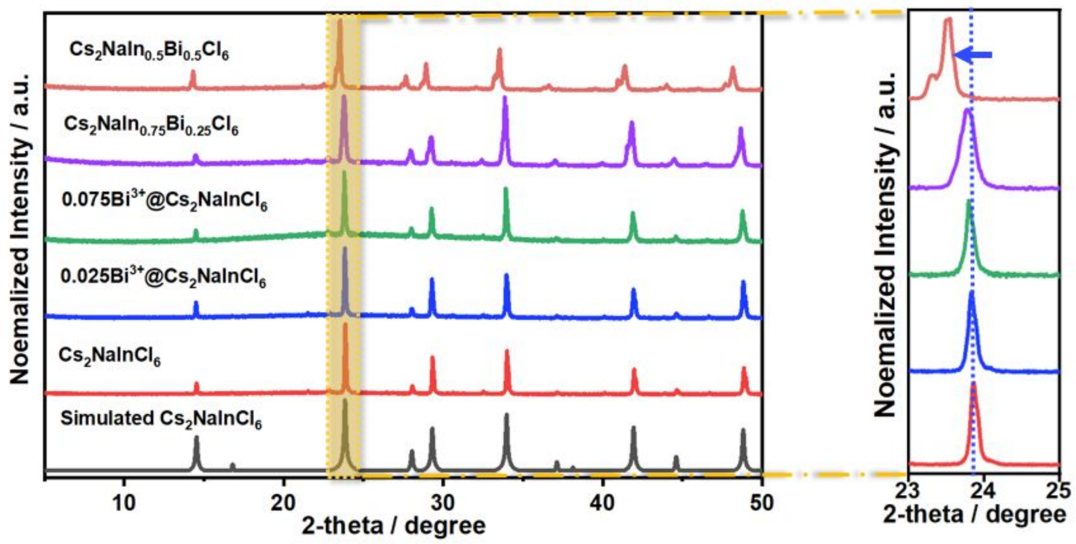

Figure S7. PXRD patterns for $Cs_2NaInCl_6$ with different Bi^{3+} doping ratios. As shown on the left, the low doping ratio of Bi^{3+} does not change the structure of $Cs_2NaInCl_6$; however, with the increased Bi^{3+} doping ratio, the peaks at 23°~25° significantly shift.

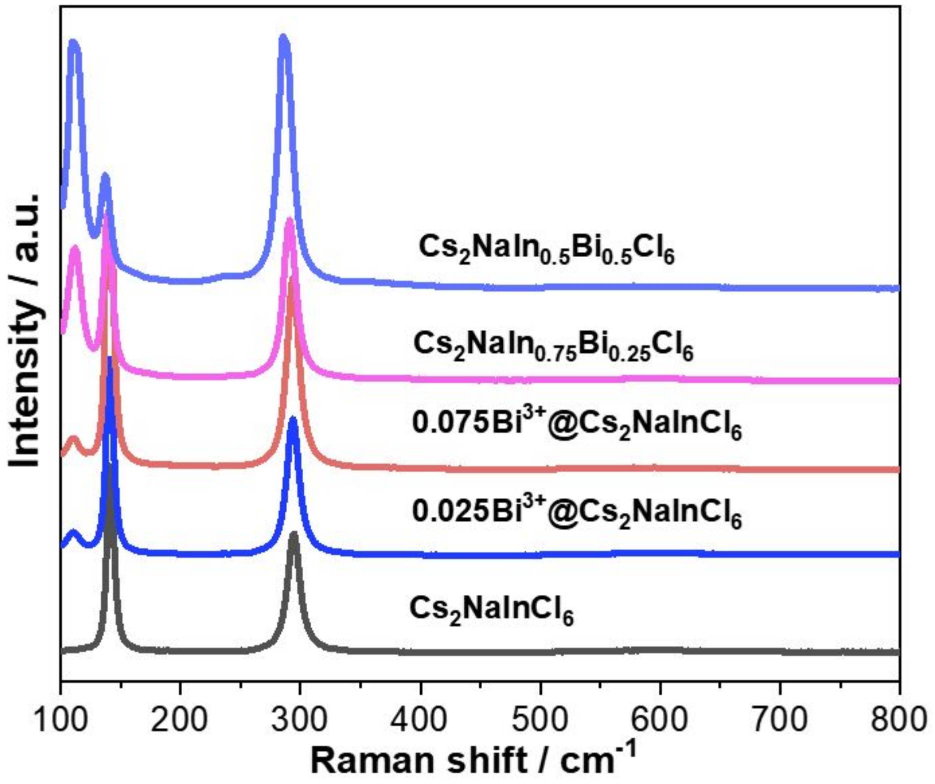

Figure S8. Raman spectra for $Cs_2NaInCl_6$ with different Bi^{3+} doping ratios. The peaks at 290 cm^{-1} are the A_{1g} stretching vibration of the $[InCl_6]^{3-}$ group.

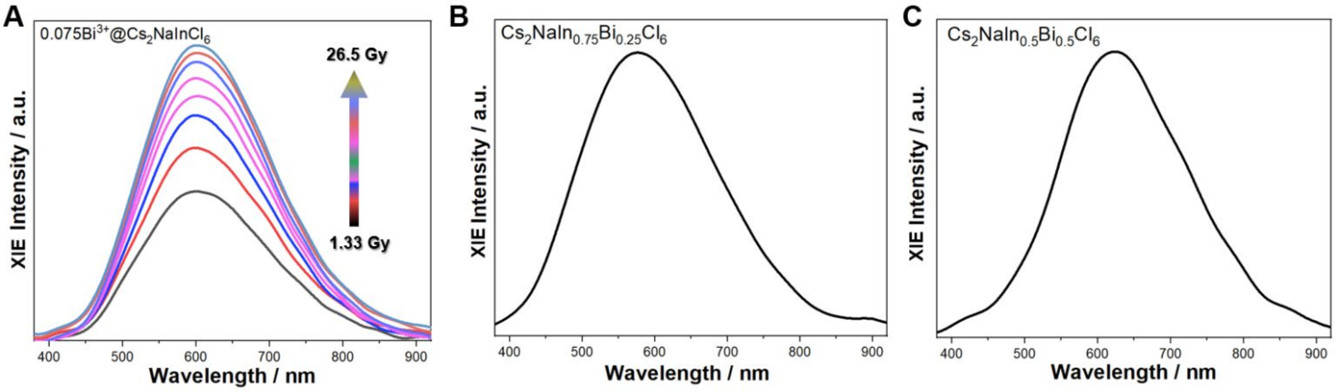

Figure S9. (A) XIE spectra of $0.025Bi^{3+}@Cs_2NaInCl_6$ at a dose rate of 26.5 Gy/min. (B) XIE spectrum of $Cs_2NaIn_{0.75}Bi_{0.25}Cl_6$ at a dose rate of 26.5 Gy/min. (C) XIE spectrum of $Cs_2NaIn_{0.5}Bi_{0.5}Cl_6$ at a dose rate of 26.5 Gy/min.

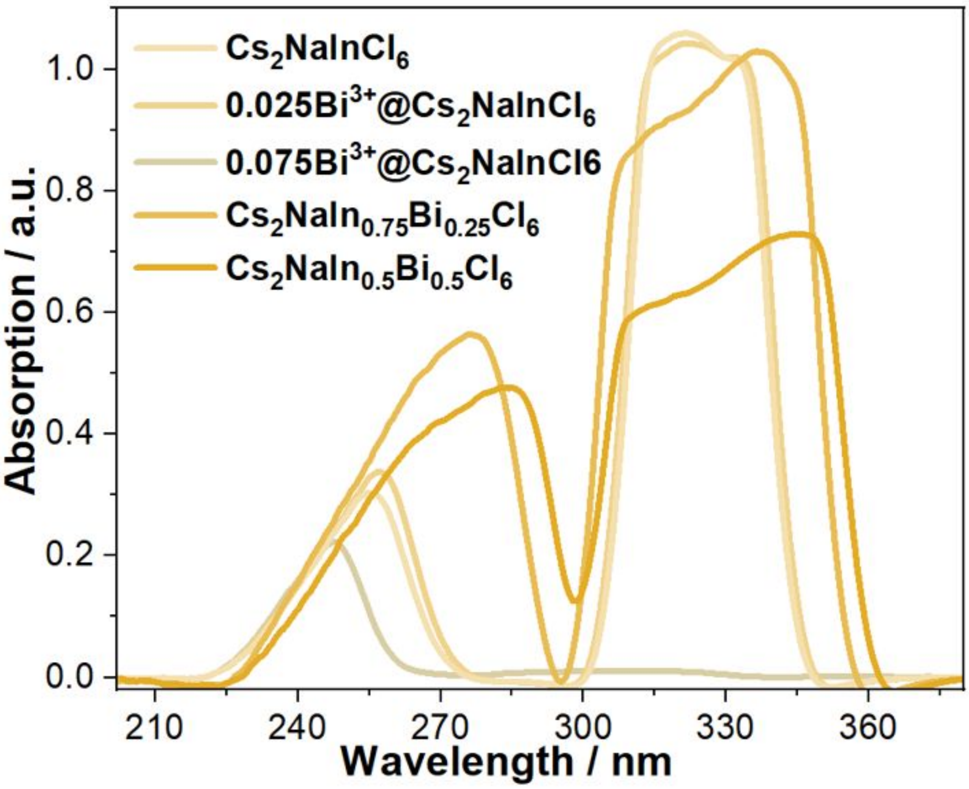

Figure S10. Absorption spectra of $Cs_2NaInCl_6$ with various Bi^{3+} doping ratios.

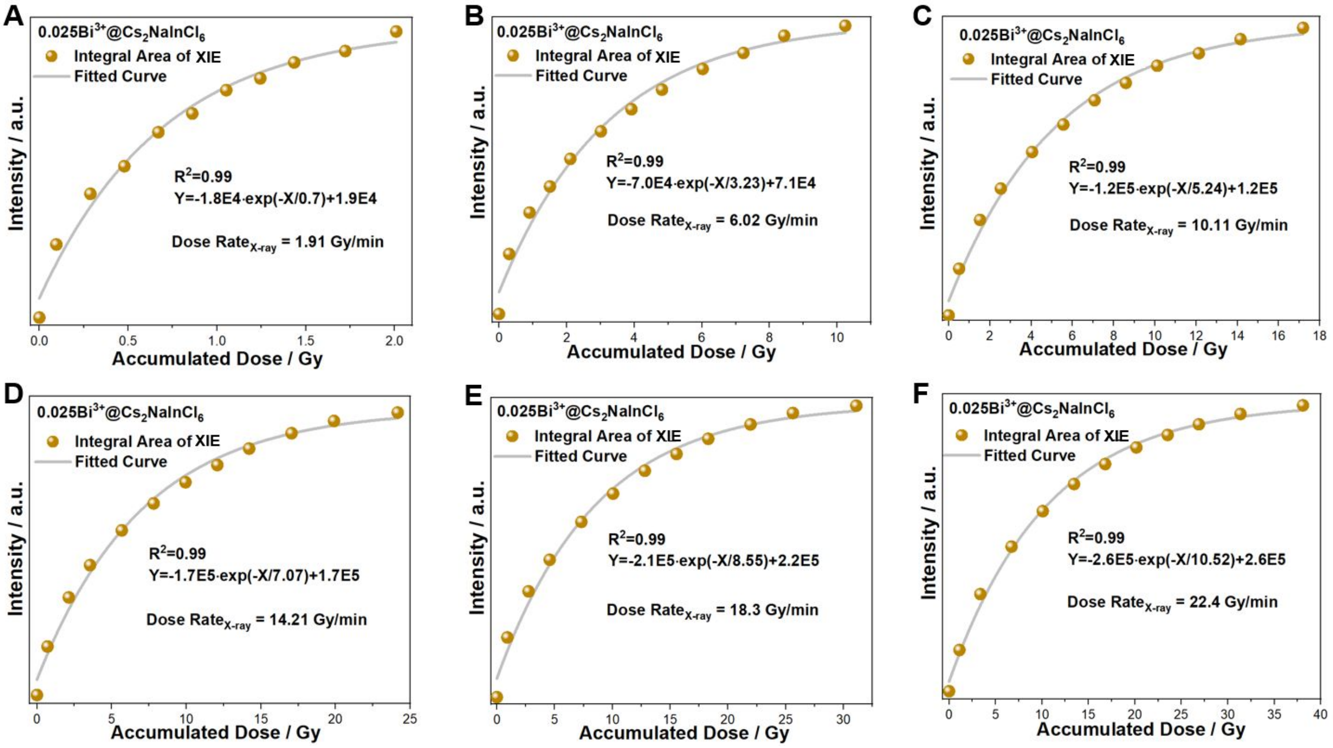

Figure S11. (A) The RL intensity of $0.025Bi^{3+}@Cs_2NaInCl_6$ as a function of accumulated dosage at a dose rate of 1.91 Gy/min, (B) 6.02 Gy/min, (C) 10.11 Gy/min, (D) 14.21 Gy/min, (E) 18.3 Gy/min, (F) 22.4 Gy/min.

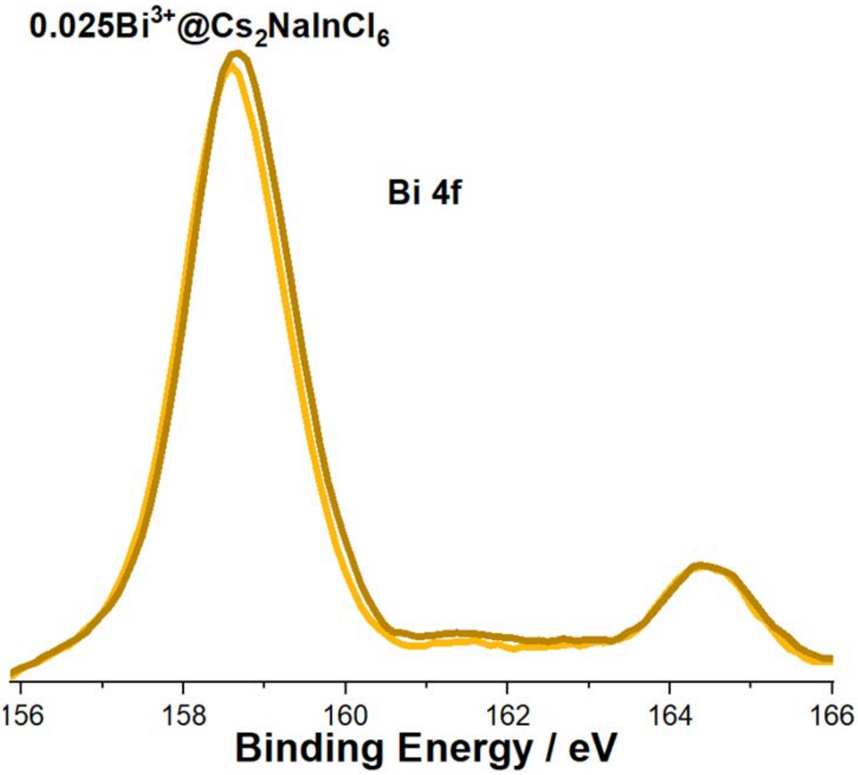

Figure S12. The change in Bi 4f in situ XPS spectra of $0.025Bi^{3+}@Cs_2NaInCl_6$ under continuous X-ray irradiation.

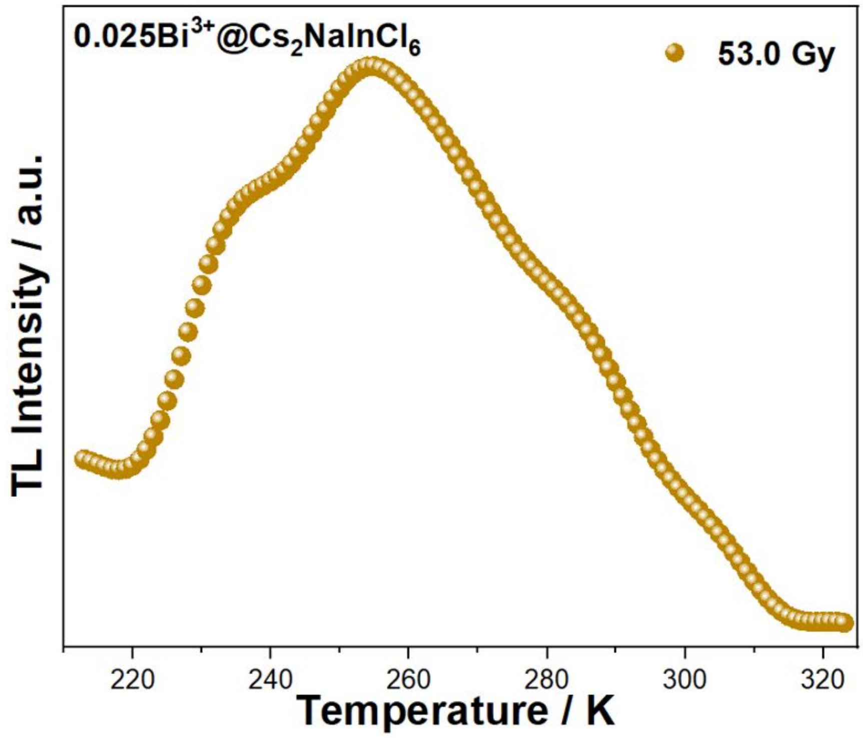

Figure S13. Thermoluminescence (TL) curve of 0.025Bi^{3+}@$Cs_2NaInCl_6$ after 53.0 Gy X-ray irradiation at 213 K.

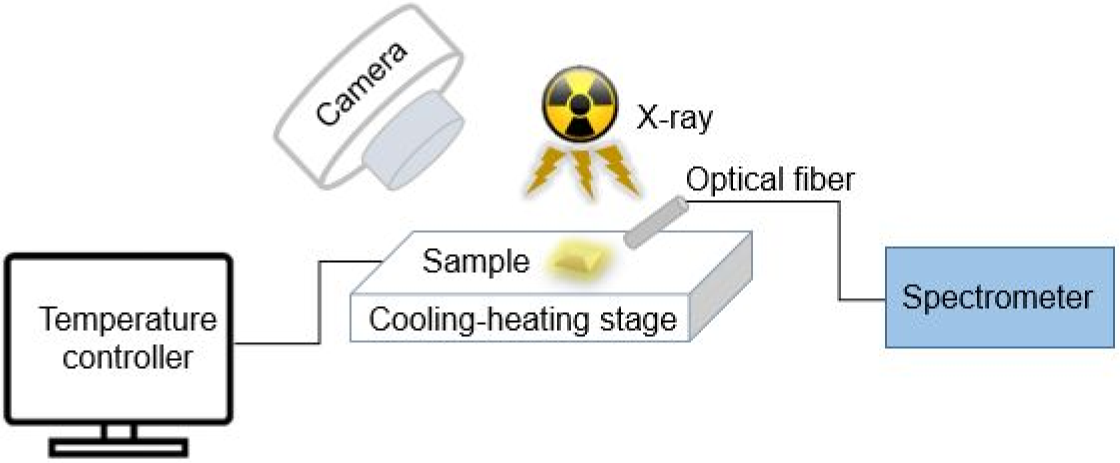

Figure S14. Schematic illustration of radioluminescence monitoring and in situ thermoluminescence collection.

Table S1. Quantitative values for the afterglow of $0.005Ag^{+}@Cs_2NaInCl_6$ irradiated with incremental X-ray dosages.

**Accumulated Dose (Gy)**	**Afterglow (s)**
**8.8**	**44.06**
**17.6**	**50.79**
**26.5**	**53.44**
**35.3**	**59.72**
**53.0**	**71.34**

Table S2. Trap depths of traditional dosimeter materials, $0.005Ag^{+}@Cs_2NaInCl_6$ and $0.025Bi^{3+}@Cs_2NaInCl_6$

**Dosimeters**	**Transition point of TL curve (K)**	**Trap depth (eV)**	**Reference**
Al_2O_3:C	473	0.95	*J. Alloy. Compd.* 886, 161262 (2021)
CaF_2	380	0.76	*Nucl. Instrum. Meth. A.* 959, 163561 (2020)
$CaSO_4$:Dy	493	0.99	*Radiat. Prot. Dosim.* 66, 213-216 (1996)
$0.005Ag^{+}@Cs_2NaInCl_6$	248.5	0.30	This work
$0.025Bi^{3+}@Cs_2NaInCl_6$	254	0.40	This work

Table S3. ICP–OES Results of $Cs_2NaInCl_6$ with various Bi^{3+}-doping ratios

Sample Labels	In (mg/g)	Bi (mg/g)	In (mmol/g)	Bi (mmol/g)	In (%)	Bi (%)
$0.025Bi^{3+}@Cs_2NaInCl_6$	3.0581	0.1884	0.0306	0.0009	97.13	2.87
$0.075Bi^{3+}@Cs_2NaInCl_6$	2.8356	0.2100	0.0247	0.0010	96.09	3.91
$Cs_2NaIn_{0.75}Bi_{0.25}Cl_6$	2.8627	1.6405	0.0249	0.0078	76.06	23.94
$Cs_2NaIn_{0.5}Bi_{0.5}Cl_6$	2.7068	2.2458	0.0236	0.0107	68.69	31.31

Table S4. Dose response range of traditional dosimeter materials and shallow-trap storage phosphors

Dosimeters	Dose response range (Gy)	Reference
α-Al_2O_3:C	$1.0\times10^{-6}\sim30$	*Radiat. Prot. Dosim.* 33, 119 (1990)
CaF_2：Mn	$1.0\times10^{-6}\sim1.0\times10^{4}$	*Nucl. Instrum. Methods Phys. Res., Sect. B.* 267,3337 (2009)
$CaSO_4$:Dy	$1.0\times10^{-5}\sim1.0\times10^{3}$	*Radiat Prot Dosim.* 100, 337 (2002)
$0.005Ag^{+}@ Cs_2NaInCl_6$	1.33~26.5	This work
$0.025Bi^{3+}@ Cs_2NaInCl_6$	0.08~45.05	This work

Movie S1.

The dynamic process of X-ray-induced emission and afterglow of $0.025Bi^{3+}@Cs_2NaInCl_6$.

References

(1) Perdew, J. P.; Burke, K.; Ernzerhof, M. Generalized Gradient Approximation Made Simple. *Phys. Rev. Lett.* **1996**, 77 (18), 3865-3868.

(2) Blöchl PE. Projector Augmented-wave method. *Phys. Rev. B.* **1994**, *50*, 17953.

(3) Kresse G, Furthmuller J. Efficiency of abinitio total energy calculations for metals and semiconductors using a plane-wave basis set. Comp. *Mater. Sci.* **1996**, *6*, 15-50.

(4) Kresse G, Furthmüller J. Efficient iterative schemes for abinitio total-energy calculations using a plane-wave basis set. *Phys. Rev. B.* **1996**, *54*, 11169.

(5) Monkhorst HJ, Pack JD. Special Points for Brillouin-zone Integrations. *Phys. Rev. B.* **1976**, *13*, 5188 .

(6) Kitis, G.; Chen, R.; Pagonis, V. Thermoluminescence glow-peak shape methods based on mixed order kinetics. *phys. stat. sol.* **2008**, *205* (5), 1181-1189.

(7) Yuan, L.; Jin, Y.; Su, Y.; Wu, H.; Hu, Y.; Yang, S. Optically Stimulated Luminescence Phosphors: Principles, Applications, and Prospects. *Laser Photonics Rev.* **2020**, *14* (12), 2000123.
